# Dapagliflozin Ameliorates Neural Damage in the Heart and Kidney of Diabetic Mice

**DOI:** 10.3390/biomedicines11123324

**Published:** 2023-12-16

**Authors:** Ionuț Donoiu, Georgică Târtea, Veronica Sfredel, Victor Raicea, Anca Maria Țucă, Alexandra Nicoleta Preda, Dragoş Cozma, Radu Vătășescu

**Affiliations:** 1 Department of Cardiology, University of Medicine and Pharmacy of Craiova, 200349 Craiova, Romania; ionut.donoiu@umfcv.ro (I.D.); dr.raicea.victor@gmail.com (V.R.); 2 Department of Physiology, University of Medicine and Pharmacy of Craiova, 200349 Craiova, Romania; veronicasfredel@yahoo.com (V.S.); ancabirau94@yahoo.com (A.M.Ț.); 3 Department of Cardiology, “Victor Babes” University of Medicine and Pharmacy, 2 Eftimie Murgu Sq., 300041 Timisoara, Romania; dragoscozma@gmail.com; 4 Cardio-Thoracic Pathology Department, “Carol Davila” University of Medicine and Pharmacy, 050474 Bucharest, Romania; radu_vatasescu@yahoo.com

**Keywords:** dapagliflozin, neuronal cardiorenal protection, diabetes mellitus

## Abstract

(1) Background: Measures for the control of diabetes mellitus (DM) and, especially, for the control of its complications represent a main objective of the research carried out on this disease, since both mortality and morbidity relating to DM represent real problems for the health system worldwide. The aim of our study was to evaluate nervous tissue from the heart and kidneys of mice with diabetes induced by streptozotocin (STZ) in the presence or absence of dapagliflozin (DAPA) treatment. (2) Methods: For this purpose, we used 24 C 57Bl/6 male mice, aged between 8 and 10 weeks. The mice were divided into three groups: sham (DM−), control (DM+), and treated (DM+). Diabetes mellitus was induced by injecting a single intraperitoneal dose of STZ. The duration of diabetes in the mice included in our study was 12 weeks after STZ administration; then, the heart and kidneys were sampled, and nervous tissue (using the primary antibody PGP 9.5) from the whole heart, from the atrioventricular node, and from the kidneys was analyzed. (3) Results: The density of nerve tissue registered a significant decrease in animals from the control group (DM+), to a value of 0.0122 ± 0.005 mm^2^ nerve tissue/mm^2^ cardiac tissue, compared with the sham group (DM−), wherein the value was 0.022 ± 0.006 mm^2^ nervous tissue/mm^2^ cardiac tissue (*p* = 0.004). Treatment with dapagliflozin reduced the nerve tissue damage in the treated (DM+DAPA) group of animals, resulting in a nerve tissue density of 0.019 ± 0.004 mm^2^ nerve tissue/mm^2^ cardiac tissue; a statistically significant difference was noted between the control (DM+) and treated (DM+DAPA) groups (*p* = 0.046). The same trends of improvement in nerve fiber damage in DM after treatment with DAPA were observed both in the atrioventricular node and in the kidneys. (4) Conclusions. These data suggest that dapagliflozin, when used in streptozotocin-induced diabetes in mice, reduces the alteration of the nervous system in the kidneys and in the heart, thus highlighting better preservation of cardiac and renal homeostasis, independent of any reduction in the effects of hyperglycemia produced in this disease.

## 1. Introduction

According to the latest data published by the International Diabetes Federation (IDF), approximately 10.5% of the adult population worldwide (20–79 years old) has diabetes, so approximately 540 million people worldwide live with this disease [1]. IDF projections up to the year 2045 show that there will be an increase of approximately 46% in the population with diabetes, so one in eight adults will have diabetes at that time [1].

These numbers also imply a significant increase in the complications of this disease. Thus, preventive measures for reducing the prevalence of type 2 diabetes, early diagnosis measures for all types of diabetes, and therapeutic measures used for controlling this disease and, especially, its complications have an essential role. Chronic hyperglycemia associated with diabetes causes the alteration and failure of a variety of tissues and organs, generating both microvascular complications (neuropathy, nephropathy, or retinopathy) and macrovascular complications (cardiovascular disorders) [2,3]. The evaluation of nerve changes in diabetes has aimed mainly at diabetic peripheral neuropathy (DPN) [4], but nerve changes in diabetes can constitute a true pathological pathway in the alteration of renal and cardiovascular function in diabetes mellitus, independent of the pathological mechanisms induced by hyperglycemia on renal cells or cardiomyocytes.

Sodium glucose co-transporter 2 inhibitors (SGLT2i) represent a new class of drugs that have been introduced into clinical practice over several years [5]. The SGLT2 inhibitors marketed in Europe are represented by dapagliflozin, canagliflozin, empagliflozin, and ertugliflozin [6,7,8,9]. The main mechanism of action of these drugs is represented by a reduction in the reabsorption of glucose at the renal level and an increase in its excretion in urine [10]. This mechanism offers a high safety profile for patients, being independent of the mechanism of action of insulin and, also, of the function of pancreatic beta cells, with a significant reduction in the risk of hypoglycemia [11]. Moreover, SGLT2i produce a decrease in systemic arterial pressure and body weight, thus having positive effects on the cardiovascular system, and can even have anti-inflammatory effects on the cerebral or coronary arteries [12,13,14].

Considering this background, the aim of our study was to evaluate nerve tissue from the heart and kidneys of mice with diabetes mellitus induced by streptozotocin in the presence or absence of dapagliflozin treatment.

## 2. Materials and Methods

### 2.1. Animal Experiments

This study was performed on an animal model with diabetes mellitus (DM) in which we assessed, from structural and functional points of view, the changes produced with regard to nervous tissue in the heart and kidneys. The study was analytical, experimental, and prospective. In this work, all institutional policies on the use of laboratory animals in research were followed, and the approval of the Ethics Committee of the University of Medicine and Pharmacy (UMF) in Craiova was obtained (no. 37/20.01.2023). The UMF animal facility, where our experiment took place, operates under FELASA (Federation of Laboratory Animal Science Association) accreditation. All procedures involving animals complied with the Standards Relating to the Care and Management of Experimental Animals in research and other regulations. This study complied with the relevant national, EU, and international ethics-related rules and professional codes of conduct. The experiments were carried out in accordance with the European Council Directive (86/609/EEC). Also, pathological assessments were performed in the Research Center for Microscopic Morphology and Immunology studies at UMF Craiova.

The animals used in our study were male C57BL/6 mice that were housed in a pathogen-free environment with continuous access to food and water on a 12-h light, 12-h dark schedule. Plasma glucose levels were determined when the animals were enrolled in the investigation (before the onset of diabetes) and again every two weeks after the onset of diabetes to diagnose the onset and duration of the disease. This determination was made by collecting blood from a large vein located in the tail of each animal, and a conventional glucometer (Contour Plus One, Ascensia Diabetes Care, Basel, Switzerland) was used.

To induce diabetes, male C57BL/6 mice were used, and the method represented by the intraperitoneal injection of a single dose of 150 mg/kg body weight streptozotocin (STZ, ≥98% HPLC, Sigma-Aldrich, Munich, Germany) was used.

The experiments were performed on 24 mice, aged between 8 and 10 weeks, weighing between 20 and 27 g. Before the procedures were performed, the animals were relocated from the animal care facility, where they had unrestricted access to food and water.

We randomly categorized the animals into three distinct groups, as shown below:

1. The sham group (DM−), receiving 0.9% NaCl, without streptozotocin. Diabetes did not occur in this group of animals. We evaluated the clinical changes in this group (body weight, blood sugar, and diuresis), as well as changes in the nervous system in the heart and kidneys of mice in the absence of diabetes.
2. The control group (DM+), into which a single dose of STZ, at a dose of 150 mg/kg body weight, was injected intraperitoneally. The animals in this group did not receive any therapeutic protocol. In this group of animals, biological parameters (such as weight, diuresis, polydipsia, and polyphagia) were monitored, and structural features of the nervous system at the level of the heart and kidneys were also evaluated.
3. The treated group with DM+ and dapagliflozin treatment (DM+DAPA). In these animals, after diabetes was induced by means of intraperitoneal injection of STZ and verified through the observation of elevated blood glucose levels, the therapy was given as 10 mg dapagliflozin/kg body weight administered via gastric gavage. The therapy was applied daily for 12 weeks from the onset of diabetes mellitus.

### 2.2. Tissue Preparation and Histological Analysis

Following the administration of deep anesthesia, the animals were euthanized and their hearts and kidneys were removed. The hearts were dissected and then promptly immersed in potassium chloride to be stopped in diastole. Later, these organs were fixed in 4% formalin solution for a duration of 24 h. After the 24–48 h in which the organs were fixed, they were washed for 24 h with the aim of removing the formalin fixative solution from the tissues. Afterward, they were embedded in paraffin in order to make serial sections 3 μm thick, which could then be stained and evaluated by means of an optical microscope. Following the acquisition of the tissue blocks, consecutive sections were created with a thickness of 3 μm using a high-precision automatic rotary microtome HM355S, including a transfer system of the sections to a cold-water bath and then to a hot-water bath at a temperature of 40 °C to be stretched and evened out. The sections obtained were subsequently gathered and mounted on slides with poly-L-lysine, then placed in an incubator at 60 °C and kept for 24 h. Initially, the tissues were stained via the hematoxylin–eosin technique (HE). After deparaffinization and antigen retrieval, the slices were sequentially incubated with the primary antibody PGP 9.5 for nervous system analysis (Abcam, Cambridge, UK, Recombinant Anti-PGP9.5 antibody [EPR4118]—Neuronal Marker—ab108986, dilution 1:200). Subsequently, an incubation with HRP-conjugated secondary antibody (Vector Laboratories, Newark, CA, USA, ImmPRESS^®^ HRP Goat Anti-Rabbit IgG Polymer Detection Kit, Peroxidase—MP-7451) followed, and for color visualization, diaminobenzidine was used as a chromogenic substrate, followed by counterstaining with hematoxylin. Initially, all slices were assessed using a Nikon 55i microscope (Apidrag, Bucharest, Romania), which was equipped with a 5-megapixel color cooled CCD camera and Image ProPlus AMS 9 software (Version 9, Media Cybernetics, Rockville, MD, USA). Later, all the slices were scanned using a microscope equipped with a MoticEasyScan Pro 6 scanner (Kowloon, Hong Kong) with a 20× objective, and the results were digitized by means of EasyScanner software (version 6). Image ProPlus AMS 9 software was used to carry out a quantitative analysis of the nervous tissue in the heart and kidneys (Figure S1). The whole heart slice was scanned to calculate the total area. Later, the color channel for the nervous tissue was selected, the area for this color channel and the integrated optical density (IOD) were calculated, and these were reported relative to the total area of the heart; an analogous calculation was carried out for the kidney. Separately, the atrioventricular node was manually delimited, and its area was calculated; the area and IOD were subsequently calculated for the nerve tissue in the atrioventricular node, reported as the density.

### 2.3. Statistical Analysis

Data from our study are reported as means and standard deviations. We collected the data first in Microsoft Office Excel 2010 (Microsoft Corporation, Redmond, Washington, DC, USA). Later, the data were transferred to GraphPad software (Version 9.0, San Diego, CA, USA) for statistical analysis. We applied an ANOVA to analyze the statistical differences among the means of multiple data groups. Whenever we obtained a p value less than 0.05, we regarded it as indicating a statistically significant difference between the means of the groups being compared. Moreover, *p* values of <0.05, <0.01, and <0.001 were taken to represent statistically significant, highly significant, and very highly significant differences and are marked in graphs by *, **, and ***.

## 3. Results

### 3.1. Effect of Dapagliflozin on Blood Glucose Level, Body Weight, and Diuresis

**Figure 1:**
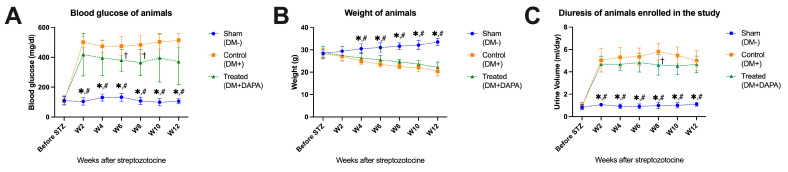
Clinical features of the animals included in our study. (**A**) Blood glucose levels. (**B**) Body weights. (**C**) Diuresis. STZ, streptozotocin; W, weeks after the intraperitoneal administration of STZ. * *p* < 0.05 vs. control (DM+), # *p* < 0.05 vs. treated (DM+DAPA), † *p* < 0.05 vs. control; two-way ANOVA, Tukey’s multiple comparison.

The blood glucose level was assessed one day before the intraperitoneal administration of STZ and then again every 2 weeks until the animals were euthanized. The same schedule was also applied regarding body weight and diuresis assessments. The blood glucose levels of the animals were similar across the groups before STZ administration. Two weeks after the STZ injection, in the control (DM+) animal group, blood glucose was at a level of 502.12 ± 38.94 mg/dL; by comparison, the group that was treated with dapagliflozin recorded a blood glucose level of 418.12 ± 142.84 mg/dL, a nonsignificant difference (*p* = 0.298). However, these two groups had very highly significantly different means from the sham group (DM−), wherein the average blood glucose was 104.37 ± 24.80 mg/dL (*p* < 0.001). The same observations were maintained throughout the animal monitoring period, with significant differences for sham (DM−) vs. control (DM+), as well as for sham (DM−) vs. treated (DM+DAPA); in contrast, for control (DM+) vs. treated (DM+DAPA), statistically significant differences were recorded only in the sixth and eighth weeks of treatment. The evolution of the blood glucose levels is highlighted in Figure 1A, the blood glucose averages for each group are presented in Supplementary Table S1, and the *p*-values are shown in Supplementary Table S2. Regarding the weight of the animals, before STZ administration, the weight of the animals was similar across the groups: 28.37 ± 1.68 g for the sham group (DM−), 28.25 ± 2.31 g for the control group (DM+), and 28.87 ± 2.69 g for the treated group (DM+DAPA). After STZ administration, the body weight of the animals in the sham group (DM−) increased, reaching values 12 weeks after STZ of 33.50 ± 1.60 g, while the weights of the animals in the other two groups decreased proportionally, reaching values at the end of the study of 20.37 ± 2.06 g in the control group (DM+) and 22.25 ± 2.25 in the treated group (DM+DAPA). The evolution of the body weight for the animals in our study can be found in Figure 1B, the averages of the animals’ weight are shown in Supplementary Table S3, and *p*-values are shown in Supplementary Table S4. Another clinical characteristic analyzed in our study was diuresis; the evolution of diuresis in the animals analyzed in our study is shown in Figure 1C, mean values of diuresis during monitoring are shown in Supplementary Table S5, and *p*-values are presented in Supplementary Table S6. A constant diuresis was noted for animals in the sham group (DM−), both before and after STZ administration, while animals from the control (DM+) and treated (DM+DAPA) groups showed a significantly higher diuresis after STZ administration.

### 3.2. Assessment of Whole-Heart Nervous Tissue

**Figure 2:**
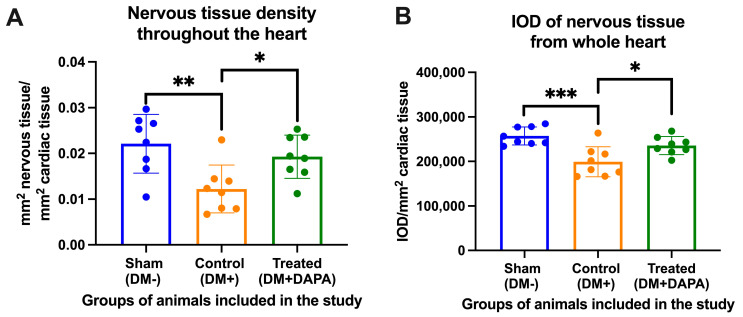
(**A**) Density of nerve tissue from the whole heart expressed as mm^2^ of nerve tissue/mm^2^ of cardiac tissue. (**B**) Integrated optical density (IOD) of nerve tissue from the whole heart expressed as IOD/mm^2^ of cardiac tissue. DM+, with diabetes mellitus; DM−, without diabetes mellitus; DAPA, dapagliflozin. *, **, or ***: *p* < 0.05, 0.01, or 0.001; one-way ANOVA, Tukey’s multiple comparison.

**Figure 3:**
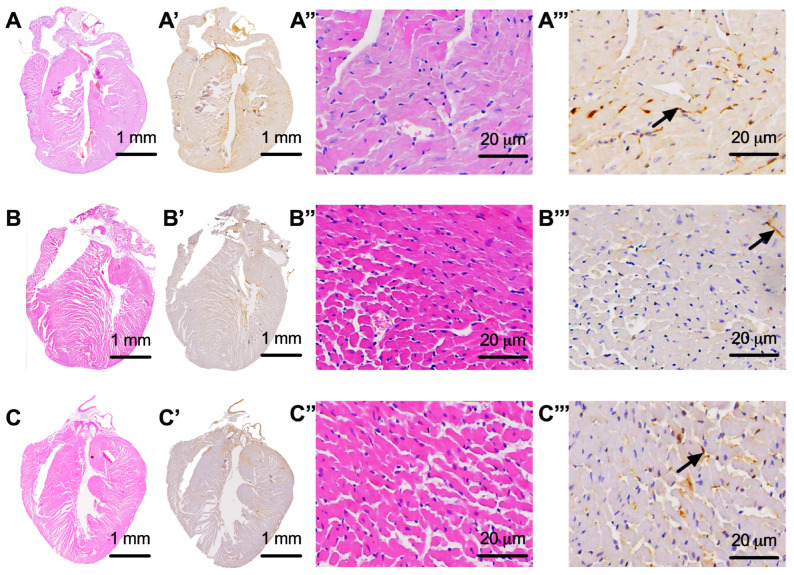
Representative images of the heart under hematoxylin–eosin staining (**A**–**C**, capital letters only), the whole heart with the nerve fibers highlighted (**A’**–**C’**, capital letters followed by ’), cardiac tissue visualized in detail under 20× objective (**A’’**–**C’’**, capital letters followed by ’’), and nervous tissue in detail from the level of the heart highlighted with black arrows (**A’’’**–**C’’’**, capital letters followed by ’’’). (**A**,**A’**,**A’’**,**A’’’**) images from non-diabetic animals that were part of the sham (DM−) group; (**B**,**B’**,**B’’**,**B’’’**) images from animals with diabetes and without treatment that were part of the control group (DM+); (**C**,**C’**,**C’’**,**C’’’**) images from diabetic animals treated with dapagliflozin that were part of the treated group (DM+DAPA).

The nerve tissue analyzed from the animals in our study presented a very low density in the control group (DM+) of only 0.0122 ± 0.005 mm^2^ of nerve tissue/mm^2^ of heart tissue, as compared with the sham group (DM−), wherein the nerve tissue density was 0.022 ± 0.006 mm^2^ of nervous tissue/mm^2^ of cardiac tissue (*p* = 0.004). The administration of dapagliflozin treatment slowed down the damage to the nervous tissue in the treated group of animals (DM+DAPA), resulting in a nervous tissue density of 0.019 ± 0.004 mm^2^ of nervous tissue/mm^2^ of cardiac tissue; a statistically significant difference between the control (DM+) and treated (DM+DAPA) groups was observed (*p* = 0.046). The area of nervous tissue and its integrated optical density in the analyzed groups are shown in Figure 2A,B, respectively, while images of the whole heart and detailed images of the nerve fibers inside the heart are shown in Figure 3.

### 3.3. Assessment of Nervous Tissue at the Level of the Atrioventricular Node

**Figure 4:**
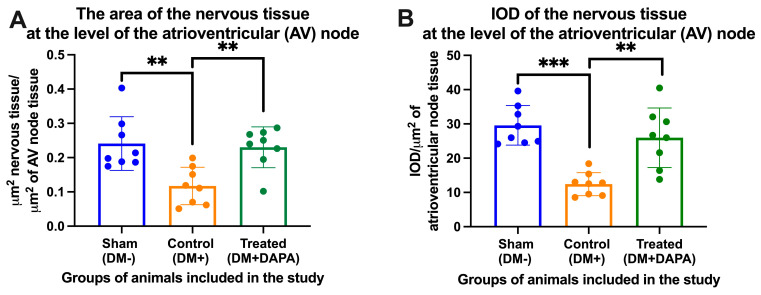
(**A**) The density of nervous tissue in the atrioventricular node (AVN) expressed as μm^2^ nervous tissue/μm^2^ of AVN. (**B**) Integrated optical density (IOD) of the nerve tissue from the whole heart expressed as IOD/μm^2^ of AVN. DM+, with diabetes mellitus; DM−, without diabetes mellitus; DAPA, dapagliflozin. ** or ***: *p* < 0.01 or 0.001; one-way ANOVA, Tukey’s multiple comparison.

**Figure 5:**
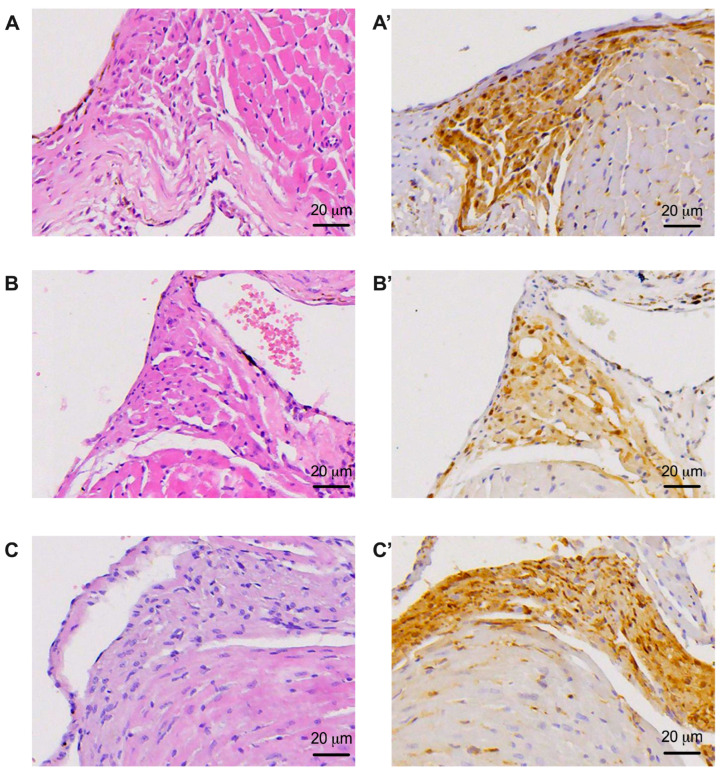
Representative images of the atrioventricular node (AVN) under hematoxylin–eosin staining (**A**–**C**, capital letters only) and of the atrioventricular node (AVN) with the nerve fibers highlighted (**A’**–**C’**, capital letters followed by ’). (**A**,**A’**) images from non-diabetic animals that were part of the sham (DM−) group; (**B**,**B’**) images from animals with diabetes and without treatment that were part of the control group (DM+); (**C**,**C’**) images from diabetic animals treated with dapagliflozin that were part of the treated group (DM+DAPA).

We manually delimited the atrioventricular node (AVN) from each heart harvested from the animals included in our study and calculated the average area of the AVN in each group of animals. We observed a reduction in the density of nerve tissue in the AVN from animals in the control group (DM+) vs. the sham group (DM−) (0.117 ± 0.054 vs. 0.2411 ± 0.078, μm^2^ nervous tissue/μm^2^ of AVN tissue). In the case of the AVN, dapagliflozin proved its neuroprotective effects: in the treated group (DM+DAPA), the average density of nerve tissue in the AVN was 0.230 ± 0.059 μm^2^ nervous tissue/μm^2^ of AV node tissue, presenting a statistically significant difference when compared with the reduced density in the control group (DM+) (*p* = 0.006). It should be mentioned that the integrated optical density recorded similar variations, and these are shown in Figure 4. Figure 5 presents images of the nerve tissue at the level of the AVN from the different groups of animals included in our study.

### 3.4. Evaluation of Nervous Tissue at the Level of the Kidneys

**Figure 6:**
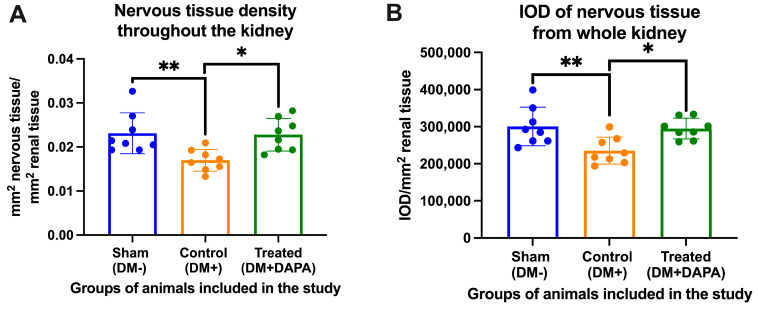
(**A**) Density of nerve tissue in the whole kidney expressed as mm^2^ of nerve tissue/mm^2^ of kidney tissue. (**B**) Integrated optical density (IOD) of the nerve tissue in the whole kidney expressed as IOD/mm^2^ of renal tissue. DM+, with diabetes mellitus; DM−, without diabetes mellitus; DAPA, dapagliflozin. * or **: *p* < 0.05 or 0.01; one-way ANOVA, Tukey’s multiple comparison.

**Figure 7:**
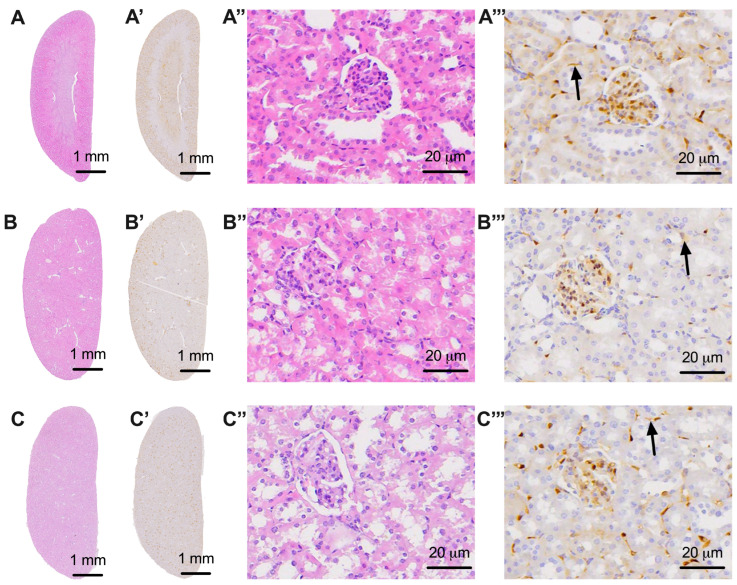
Representative images of the kidney under hematoxylin–eosin staining (**A**–**C**, uppercase letters only), the kidney with nerve fibers highlighted (**A’**–**C’**, uppercase letters followed by ’), kidney tissue visualized in detail under 20× objective (**A’’**–**C’’**, uppercase letters followed by ’’), and details of nervous tissue in the kidney highlighted with black arrows (**A’’’**–**C’’’**, capital letters followed by ’’’). (**A**,**A’**,**A’’**,**A’’’**) images from non-diabetic animals that were part of the sham (DM−) group; (**B**,**B’**,**B’’**,**B’’’**) images from animals with diabetes and without treatment that were part of the control group (DM+); (**C**,**C’**,**C’’**,**C’’’**) images from diabetic animals treated with dapagliflozin that were part of the treated group (DM+DAPA).

Like for the heart, we performed an analysis of nervous tissue in the kidney. Both the area and the integrated optical density were calculated. We observed a decrease in the density of nerve tissue in the kidneys of animals from the control group (DM+), to 0.016 ± 0.002 mm^2^ of nerve tissue/mm^2^ of kidney tissue, as compared with the sham group (DM−), wherein the density was 0.023 ± 0.004 mm^2^ of nervous tissue/mm^2^ of renal tissue (*p* = 0.009). Dapagliflozin had a protective role, resulting in a much smaller decrease in the density of nerve tissue in the kidney in animals with diabetes treated with dapagliflozin, to a value of 0.022 ± 0.003 mm^2^ of nerve tissue/mm^2^ of kidney tissue, as compared with the sham group (DM−) (*p* = 0.013). In the case of IOD, the same variations were recorded, as shown in Figure 6. Images of the nervous tissue in the kidneys are shown in Figure 7.

## 4. Discussion

Our study reports, for the first time, a protective effect of dapagliflozin on the nervous system, observed in the heart and kidneys of mice with streptozotocin-induced diabetes. Thus, there are two main directions for the discussion of this topic. The first is about the molecular mechanisms that contribute to nervous tissue damage in diabetes. Secondly, we must discuss the role of dapagliflozin in reducing this damage to the nervous system in the heart and kidneys of mice with diabetes mellitus.

The molecular mechanisms that contribute to neuronal damage in diabetes are multiple [15]. One well-studied pathway is the polyol pathway. In diabetes, excess glucose is metabolized via the polyol pathway, aldose reductase (AR) reduces glucose to sorbitol with the help of nicotinamide adenine dinucleotide phosphate (NADPH), and then sorbitol is oxidized to fructose by sorbitol dehydrogenase in a process involving nicotinamide adenine dinucleotide (NAD+). Thus, reserves of NADPH are depleted, with a downregulation of GSH, which causes damage to the endothelial cells via the impairment of vasodilatation mediated by nitric oxide. This affects the vascularization of nerve fibers [15,16]. Persistent hyperglycemia generates an increased concentration of fructose-6-phosphate, which is transformed into glucosamine-6-phosphate via the hexosamine pathway, followed by the production of N-acetyl glucosamine (GlcNAc) [17]. GlcNAc causes oxidative stress, which leads to functional deficit in pancreatic beta cells. Moreover, this pathological pathway results in increased levels of hydrogen peroxide, producing additional oxidative stress that affects the neuronal environment [15]. Besides hexosamine, the generation of advanced glycation end products (AGEs) is another way by which nerves can be affected. Increased concentrations of AGEs were identified at the level of peripheral nerves in rats with streptozotocin-induced diabetes mellitus. Furthermore, AGEs can cause inflammation by up-regulating nuclear factor-kb (NF-kB) and apoptosis by increasing inflammatory cytokines such as tumor necrosis factor alpha (TNF-alpha) and interleukin-1 beta [18,19]. These pathways have a significant impact on neuronal function and the neuronal environment [20]. In addition to these, pathways such as protein kinase C, poly adenosine phosphate ribose polymerase, and mitogen-activated protein kinase (MAPK) can play a prominent role in neuronal disease [21,22,23].

Data from the EMPA-REG OUTCOME and CANVAS trials show that SGLT2 inhibition has beneficial effects on renal and cardiovascular outcomes; however, the mechanisms by which all this is possible have not been elucidated [24,25,26]. The cardiorenal protection conferred via a neuronal mechanism by SGLT2i obligatorily involves the two subdivisions of the autonomic nervous system: the sympathetic and parasympathetic arms. In our study, the autonomic nervous system as a whole was evaluated at the renal and cardiac levels. Prior studies have evaluated either the sympathetic arm or the parasympathetic arm in animals treated with SGLT2i. Regarding the sympathetic autonomic nervous system, the studies are more numerous [27]. The sympathetic nervous system (SNS) up-regulates SGLT2 protein levels, and their blockade has been shown to have renal and cardiac protective properties in obese HFD-fed mice [28]. Compared with our study where the blood glucose level was very high in mice with diabetes mellitus (by 300–400%) compared with mice without diabetes, in the study cited above an animal model was used in which the level of blood glucose was much less increased (by only 20–30%) compared with mice with normal food, and was secondary to the production of metabolic syndrome by high-fat diet (HFD) administration. Moreover, in the HFD-fed mouse model they observed an increase in the sympathetic innervation (by increasing the expression of tyrosine hydroxylase) compared with mice with a normal diet, both in the kidneys and in the heart [28]. This increase can be partially explained by obesity, but other mechanisms are also possible. Treatment of HFD mice with DAPA showed a reduction of tyrosine hydroxylase, respectively noradrenaline [28]. In our study, the entire nerve area (both sympathetic and parasympathetic) was evaluated by PGP 9.5 and the very high levels of glucose in the blood of animals that received STZ determined a decrease in this area, compared with animals without diabetes. This decrease can be explained through neuronal damage induced by the molecular mechanisms discussed at the beginning of this section. This decrease was lower in animals with diabetes mellitus induced by STZ, treated with DAPA. The most plausible hypothesis for our study was represented by the decrease in the blood glucose level, which led to the reduction of the molecular mechanisms of neuronal damage. Moreover, a recent study even showed crosstalk between SNS activity and SGLT2, since chemical denervation of the SNS promotes decreased SGLT2 expression, and hypertensive BPH/2J mice treated with the SGLT2 inhibitor DAPA showed reduced sympathetic innervation [29]. Another study reported a decrease in heart rate (HR) in rats treated with dapagliflozin, but the mechanism was not elucidated [30]. Moreover, treatment with ertugliflozin, another STGT2i, has been shown to have an infarct-limiting effect via activation of the vagus nerve and M3-cholinoreceptors [31]. Saleh S et al. showed in a recent study that treatment with DAPA reduced systolic and diastolic blood pressure but increased heart rate in rats with DM, as compared with animals with DM that did not receive treatment [32]. Moreover, treatment with DAPA in mice with DM significantly reduced 24 h proteinuria, as compared with mice with DM but without treatment [33].

The choice of analyzing the nervous system at both the cardiac and renal levels was justified both by the synergistic influences of the nervous system in physiological conditions and by the cardiorenal alterations that occur in diabetes mellitus and in other pathologies [34,35,36].

### Limitations of This Study

The main limitation of our study was the absence of an evaluation of sympathetic and parasympathetic nerve fibers in the heart and kidneys of the mice in the analyzed groups. Another limitation of our study is represented by the neuronal analysis at the renal level. PGP 9.5 is an immunomarker that is expressed not only by central or peripheral neurons but also by certain metabolically active epithelial cells at the renal level [37]; therefore, the neuronal analysis at the renal level had a lower relevance than that at the heart level. However, the observations at the renal level between the groups of animals studied were like those at the heart level. Lastly, these observations were made on an animal model of mice with diabetes, but they must be translated to the human heart and kidneys to see whether the results are also valid in humans.

## 5. Conclusions

Dapagliflozin, when used in mice with streptozotocin-induced diabetes, ameliorates the deterioration of the nervous system in the kidneys and heart, suggesting a better preservation of cardiac and renal homeostasis, independent of any reduction in the effects of hyperglycemia produced in this disease.

## Data Availability

Data are contained within the article or available upon request from the corresponding authors.

## Supplementary Materials

The following supporting information can be downloaded at: https://www.mdpi.com/article/10.3390/biomedicines11123324/s1. Figure S1. Representative pictures with imaging analysis using Image ProPlus AMS 9 software. (A) Heart area calculation in mm^2^. (B) Selecting the appropriate color channel for PGP 9.5. (C) Area counting for the corresponding color channel. Table S1: Mean and standard deviation (SD) of blood glucose for animals enrolled in our study. Table S2: Two-way ANOVA and multiple comparisons of blood glucose between groups enrolled in our study before and in the weeks after the occurrence of diabetes mellitus. Table S3: Mean and standard deviation (SD) of the body weights of animals enrolled in our study. Table S4: Two-way ANOVA and multiple comparisons of body weight between groups enrolled in our study before and in the weeks after the occurrence of diabetes mellitus. Table S5: Mean and standard deviation (SD) of diuresis in the animals enrolled in our study. Table S6: Two-way ANOVA and multiple comparisons of diuresis between groups enrolled in our study before and in the weeks after the occurrence of diabetes mellitus.
