# Supplementary material for: Dapagliflozin Ameliorates Neural Damage in the Heart and Kidney of Diabetic Mice
Source: Biomedicines. 2023 Dec 16;11(12):3324. doi: 10.3390/biomedicines11123324 (PMC10741899; doi:10.3390/biomedicines11123324)
Supplement: Supplementary file 1 [file biomedicines-11-03324-s001.zip › biomedicines-2753838-supplementary.pdf]

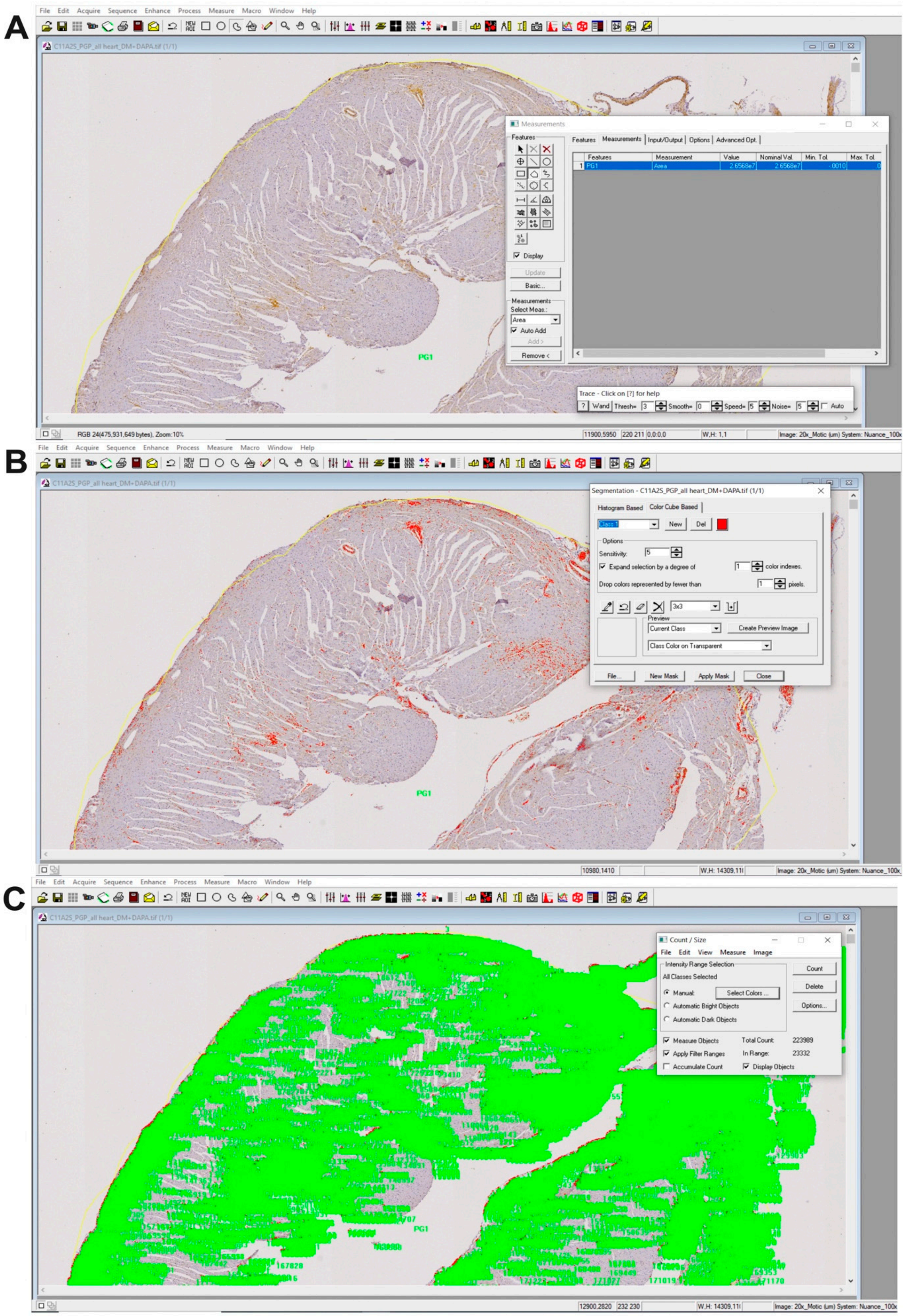

Figure S1. Representative pictures with imaging analysis using Image ProPlus AMS 9 software. A - heart area calculation in mm2. B - selecting the appropriate color channel for PGP 9.5. C - Area counting for the corresponding color channel.

Table S1. Mean and standard deviation of mean (SD) of blood glucose for animals enrolled in our study.

	Sham (DM-)		Control (DM+)		Treated (DM+DAPA)	
	Mean	SD	Mean	SD	Mean	SD
Before STZ	109,63	27,06	104,63	27,34	117,50	23,60
W2	104,38	24,81	502,13	38,94	418,13	142,85
W4	130,63	23,69	473,63	41,89	396,00	118,03
W6	132,88	26,61	474,63	62,95	380,88	74,37
W8	107,88	25,49	483,75	60,06	364,88	87,96
W10	99,25	24,36	504,00	49,14	396,63	162,33
W12	105,00	17,41	513,38	45,53	370,63	154,28

Table S2. 2way ANOVA multiple comparisons for blood glucose between groups enrolled in our study before and in the weeks after the occurrence of diabetes mellitus.

Tukey's multiple comparisons test	Summary	Adjusted P Value
Before STZ		
Sham (DM-) vs. Control (DM+)	ns	0,9286
Sham (DM-) vs. Treated (DM+DAPA)	ns	0,8116
Control (DM+) vs. Treated (DM+DAPA)	ns	0,5842
W2		
Sham (DM-) vs. Control (DM+)	****	<0,0001
Sham (DM-) vs. Treated (DM+DAPA)	***	0,001
Control (DM+) vs. Treated (DM+DAPA)	ns	0,298
W4		
Sham (DM-) vs. Control (DM+)	****	<0,0001
Sham (DM-) vs. Treated (DM+DAPA)	***	0,0008
Control (DM+) vs. Treated (DM+DAPA)	ns	0,2405
W6		
Sham (DM-) vs. Control (DM+)	****	<0,0001
Sham (DM-) vs. Treated (DM+DAPA)	****	<0,0001
Control (DM+) vs. Treated (DM+DAPA)	*	0,0419
W8		
Sham (DM-) vs. Control (DM+)	****	<0,0001
Sham (DM-) vs. Treated (DM+DAPA)	***	0,0001
Control (DM+) vs. Treated (DM+DAPA)	*	0,0203
W10		
Sham (DM-) vs. Control (DM+)	****	<0,0001
Sham (DM-) vs. Treated (DM+DAPA)	**	0,003
Control (DM+) vs. Treated (DM+DAPA)	ns	0,2311
W12		
Sham (DM-) vs. Control (DM+)	****	<0,0001
Sham (DM-) vs. Treated (DM+DAPA)	**	0,0043
Control (DM+) vs. Treated (DM+DAPA)	ns	0,082

Table S3. Mean and standard deviation of mean (SD) of body weight of the animals enrolled in our study.

	Sham (DM-)		Control (DM+)		Treated (DM+DAPA)	
	Mean	SD	Mean	SD	Mean	SD
Before STZ	28,38	1,69	28,25	2,31	28,88	2,70
W2	29,38	2,00	27,00	2,14	27,38	2,39
W4	30,50	2,20	25,00	1,51	26,38	2,26
W6	31,00	2,14	23,50	1,20	25,50	2,00
W8	31,63	1,51	22,50	0,93	24,63	1,19
W10	32,13	2,03	22,00	1,69	23,63	1,51
W12	33,50	1,60	20,38	2,07	22,25	2,25

Table S4. 2way ANOVA multiple comparisons for body weight between groups enrolled in our study before and in the weeks after the occurrence of diabetes mellitus.

Tukey's multiple comparisons test	Summary	Adjusted P Value
Before STZ		
Sham (DM-) vs. Control (DM+)	ns	0,9916
Sham (DM-) vs. Treated (DM+DAPA)	ns	0,8976
Control (DM+) vs. Treated (DM+DAPA)	ns	0,8737
W2		
Sham (DM-) vs. Control (DM+)	ns	0,0895
Sham (DM-) vs. Treated (DM+DAPA)	ns	0,2007
Control (DM+) vs. Treated (DM+DAPA)	ns	0,9416
W4		
Sham (DM-) vs. Control (DM+)	***	0,0002
Sham (DM-) vs. Treated (DM+DAPA)	**	0,0064
Control (DM+) vs. Treated (DM+DAPA)	ns	0,3574
W6		
Sham (DM-) vs. Control (DM+)	****	<0,0001
Sham (DM-) vs. Treated (DM+DAPA)	***	0,0003
Control (DM+) vs. Treated (DM+DAPA)	ns	0,0777
W8		
Sham (DM-) vs. Control (DM+)	****	<0,0001
Sham (DM-) vs. Treated (DM+DAPA)	****	<0,0001
Control (DM+) vs. Treated (DM+DAPA)	**	0,004
W10		
Sham (DM-) vs. Control (DM+)	****	<0,0001
Sham (DM-) vs. Treated (DM+DAPA)	****	<0,0001
Control (DM+) vs. Treated (DM+DAPA)	ns	0,1419
W12		
Sham (DM-) vs. Control (DM+)	****	<0,0001
Sham (DM-) vs. Treated (DM+DAPA)	****	<0,0001
Control (DM+) vs. Treated (DM+DAPA)	ns	0,2274

Table S5. Mean and standard deviation of mean (SD) of diuresis of the animals enrolled in our study.

	Sham (DM-)		Control (DM+)		Treated (DM+DAPA)	
	Mean	SD	Mean	SD	Mean	SD
Before STZ	0,8375	0,226385	0,975	0,296407	0,975	0,249285
W2	1,0625	0,150594	5,0375	1,048724	4,6875	0,712014
W4	0,925	0,198206	5,3125	0,893528	4,675	0,533854
W6	0,9125	0,223207	5,35	0,781939	4,825	0,843039
W8	0,9875	0,274838	5,8125	0,737636	4,625	0,905144
W10	1	0,226779	5,4625	0,763334	4,5375	0,792712
W12	1,1	0,169031	5,0125	0,904651	4,6625	0,744384

Table S6. 2way ANOVA multiple comparisons for diuresis between groups enrolled in our study before and in the weeks after the occurrence of diabetes mellitus.

Tukey's multiple comparisons test	Summary	Adjusted P Value
Before STZ		
Sham (DM-) vs. Control (DM+)	ns	0,5642
Sham (DM-) vs. Treated (DM+DAPA)	ns	0,498
Control (DM+) vs. Treated (DM+DAPA)	ns	>0,9999
W2		
Sham (DM-) vs. Control (DM+)	****	<0,0001
Sham (DM-) vs. Treated (DM+DAPA)	****	<0,0001
Control (DM+) vs. Treated (DM+DAPA)	ns	0,7211
W4		
Sham (DM-) vs. Control (DM+)	****	<0,0001
Sham (DM-) vs. Treated (DM+DAPA)	****	<0,0001
Control (DM+) vs. Treated (DM+DAPA)	ns	0,2355
W6		
Sham (DM-) vs. Control (DM+)	****	<0,0001
Sham (DM-) vs. Treated (DM+DAPA)	****	<0,0001
Control (DM+) vs. Treated (DM+DAPA)	ns	0,4228
W8		
Sham (DM-) vs. Control (DM+)	****	<0,0001
Sham (DM-) vs. Treated (DM+DAPA)	****	<0,0001
Control (DM+) vs. Treated (DM+DAPA)	*	0,0317
W10		
Sham (DM-) vs. Control (DM+)	****	<0,0001
Sham (DM-) vs. Treated (DM+DAPA)	****	<0,0001
Control (DM+) vs. Treated (DM+DAPA)	ns	0,0775
W12		
Sham (DM-) vs. Control (DM+)	****	<0,0001
Sham (DM-) vs. Treated (DM+DAPA)	****	<0,0001
Control (DM+) vs. Treated (DM+DAPA)	ns	0,6825
